# Genistein Ameliorated Vascular Endothelial Growth Factor-A (VEGF-A) and Estrogen Receptor-Alpha (ER-*α*) in Endometriosis Mice Model, *In Vivo* and *In Silico*

**DOI:** 10.1155/2024/5338212

**Published:** 2024-01-24

**Authors:** Sutrisno Sutrisno, Maharani Maharani

**Affiliations:** ^1^Department of Obstetrics and Gynecology, Faculty of Medicine, University of Brawijaya, Saiful Anwar General Hospital, Malang, East Java, Indonesia; ^2^Magister of Midwifery, Faculty of Medicine, University of Brawijaya, Malang, East Java, Indonesia; ^3^Department of Midwifery, Polytechnic of Health-Ministry of Health, Aceh, Indonesia

## Abstract

Endometriosis (EM) is a gynecological disorder that causes morbidity in women and is characterized by endometrial tissue in the uterus cavity. This study investigated the mechanism of genistein in the VEGF-A and ER-*α* expression through in vivo and in silico approaches. An in vivo study was conducted by thirty-six mice that were divided into six groups including control, EM, and EM treatment with genistein with the doses of 1.3, 1.95, 2.6, and 3.25 mg/day for 14 days. Peritoneal tissues with lesions were collected and analyzed by immunohistochemistry to measure the VEGF-A and ER-*α* expression. The data were analyzed using a statistical approach using one-way ANOVA followed by Tukey HSD test with a significant value *p* < 0.05. In silico study was conducted for investigating the inhibition mechanism of genistein in VEGF-A and ER-*α* protein. Genistein significantly reduced the VEGF-A and ER-*α* expression with the optimum dose of 3.25 mg/day. Molecular docking showed that genistein inhibited VEGF-A in several active site residues of VEGF-A, also blocked the ER-*α* protein in estradiol binding sites. This study concluded that genistein prevented endometriosis by performing the antiangiogenic activity and showed a similar function to estradiol.

## 1. Introduction

Endometriosis is an estrogen dependence disorder characterized by chronic pelvic pain, dysmenorrhea, dyspareunia, dyschezia, and endometrial tissue in the uterus cavity [1, 2]. The prevalence of endometriosis was reported 2% and 10% in the general population, 50% of them in infertile patients, and 60% of them in chronic pelvic pain [1, 3, 4]. Several treatment management strategies have been developed to reduce endometriosis occurrences [1, 5, 6]. Contraceptive therapy reduced endometriosis-associated pain by inhibiting hypothalamus-ovarian axis and repressing ovulatory functions [7, 8]. Laparoscopic management is also used to exclude malignancy and prevent endometriosis expansion [9] However, laparoscopic procedures have reported negative effects on several female patients. Some treatments have been reported to suppress the endometriosis, including hormonal therapy, aromatase inhibitors, angiogenic inhibitors, immunomodulators, and antioxidant compounds [6]. The mechanism of endometriosis is complex and depicted in several pathways.

In endometriosis-dependent estrogen pathway, estrogen activates peritoneal macrophages to stimulate proinflammatory cytokines, including tumor necrosis factor (TNF) and interleukin-1*β* (IL-1*β*). Simultaneously, estrogen also induces proinflammatory cytokine production and activates transcription factors (NF-kB, AP-1) to produce inflammatory cytokines and induce angiogenesis via VEGF and nitric oxide (NO) accumulation. Besides that, activated transcription factors promote cell movement by repressing matrix metalloproteinase activities. Degrading matrix metalloproteinase (MMP) induces ectopic endometrial cell invasion, adhesion, and tissue remodelling [10, 11]. Endometriosis is also caused by the imbalancing of oxidative stress and antioxidants. High levels of oxidative stress promote endometriosis [11]. Therefore, several studies revealed some antioxidant compounds performed endometriosis-preventing mechanism. Those compounds involved vitamin C, vitamin E, curcumin, resveratrol, flavonoids, and epigallocatechin [1, 5, 11, 12].

Dietary isoflavones for women prevent breast cancer, induce fertility in males and females, and decrease menopausal symptoms and others [13]. Genistein is an isoflavonoids compound with the IUPAC name 5,7-dihydroxy-3-(4-hydroxyphenyl) chromen-4-one. Genistein was found in soybean and soybean products, involving tofu, tempeh, soybean milk, and others [13, 14]. Genistein has several biological activities, including high antioxidants, reducing nitric oxide (NO), exhibiting apoptosis-induced pathways, decreasing atherogenic conditions, and hypercholesterolemia [13, 15, 16]. The mechanism of genistein in endometriosis therapy remains unclear. Therefore, the present study provided the effect of genistein at various concentrations on the VEGF-A and ER-*α* expression. The mechanism of VEGF-A and ER-*α* inhibition was also investigated by the in silico study.

## 2. Materials and Methods

### 2.1. *In Vivo* Experimental Analysis

#### 2.1.1. Experimental Endometriosis Mice Model

All animal works in this study were approved by the Health Research Ethics Committee, Faculty of Medicine, Brawijaya University, Malang, Indonesia (502/EC/KEPK/09/2014). Thirty-six female mice, 2-3 months old, 20–30 gram body weight, were provided by the Reproductive Physiology Embryology Laboratory, Faculty of Veterinary Medicine, Airlangga University, Surabaya. All mice were divided into control, EM, and EM mice with genistein treatment. Endometriosis mice models were conducted by injecting 0.2 mL cyclosporin A for mice immunodeficiency. The myometrium and endometrium tissues from patients with adenomyosis were collected and kept in phosphate buffer saline (PBS). The tissues were sliced into 0.5 cm, washed with PBS, and then centrifuged at 3000 rpm at 4°C for 10 minutes. 0.1 mL of supernatant was injected into the peritoneal cavity. To induce endometriosis in mice, 0.1 mL of ethinyl estradiol was injected intramuscularly on days 1 and 5. A positive EM mice model was shown 15 days after estradiol injections [17].

#### 2.1.2. Genistein Treatment and Immunohistochemical Analysis

Genistein (Tokyo Chemical Industry, Japan) was dissolved in sesame oil to make 1 gr/mL. Genistein was orally administered for 14 days in four treatment groups. Those groups were EM with 1.3 mg/day of genistein (EM + 1.3), EM with 1.95 mg/day of genistein (EM + 1.95), EM with 2.6 mg/day of genistein (EM + 2.6), and EM with 3.25 mg/day of genistein (EM + 3.25). Anesthesia with 100 mg/kg ketamine and 10 mg/kg xylazine was used for all mice surgeries. The peritoneal tissue was collected for immunohistochemical analysis. The peritoneal tissue with the lesion was sliced to 0.5 cm thickness and soaked in 10% formalin for 24 h. Then, the tissue was dehydrated using a serial dilution of alcohol and cleared using xylol. The lesion tissue was embedded in paraffin block by automatic tissue embedding apparatus tissue-Tek. The lesion tissue was sliced into 6 *µ*m thickness, followed by deparaffinization. The standard immunohistochemistry procedure assessed the VEGF-A and ER-*α* expression in peritoneal tissue. Image capturing was conducted using an electric light microscope Nikon H600L with a digital camera DS F12 300MPx.

### 2.2. Statistical Analysis

Animal experimental data were obtained using SPSS version 15.0 software. One-way ANOVA analysis was conducted to compare groups, and multiple comparisons were done using Tukey's post hoc test with *p* value <0.05.

### 2.3. In Silico Analysis

#### 2.3.1. Ligands and Proteins Retrieval

Three-dimensional structures of genistein and estradiol were retrieved from the PubChem database with the number identities CID_5280961 and CID_5757, respectively. Vascular endothelial growth factor-A (VEGF-A) and estrogen receptor-*α* were obtained from a protein data bank with ID 3QTK and 6VPK [18]. The protein structure was prepared by cavity identification using Molegro Virtual Docker version 5.0 for further docking analysis [19].

#### 2.3.2. Molecular Docking and Visualization

Prepared VEGF-A and estrogen receptor-*α* proteins were docked with genistein using Molegro Virtual Docker version 5.0 software [19]. Estradiol or estrogen was used as a native ligand for ER-*α* protein. Docking was conducted in MolDock Grid Score 0.3A, RMSD less than 2.0, and ten times docking running. The binding energies of ligand-protein complex interactions were summarized from the MolDock Score, MolDock Grid Score, and rerank score and then averaged from 10 docking repetitions [18, 19]. The complex interactions were visualized by PyMol version 2.2. and Discovery Studio version 21.1.1.

## 3. Results

**Figure 1:**
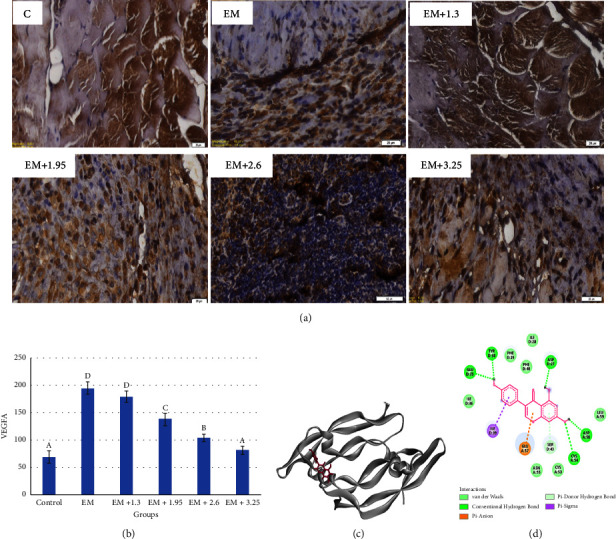
Immunohistochemical profiles and molecular interaction of genistein in endometriosis mice model. (a) Immunohistochemistry profiles, (b) VEGF-A expression levels, (c) binding sites of genistein in VEGF-A protein, and (d) two-dimensional structure of genistein with VEGF-A.

The VEGF-A expression in peritoneal tissue is illustrated in Figure 1(a). Control or normal mice revealed lower VEGF-A expression, and increased VEGF-A was exposed in the endometriosis mice model. The administration of genistein at various concentrations decreased VEGF-A in endometriosis mice models. The optimum concentration of genistein to improve the VEGF-A levels was 3.25 mg/day (Figure 1(b)). Genistein was bound to VEGF-A in several binding sites of VEGF-A and generated binding energy −274.3 kJ/mol (Table 1). The interaction types involved hydrogen bonds and electrostatic and hydrophobic interactions.

Interestingly, van der Waals was also shown in the genistein-VEGF-A complex (Figures 1(c) and 1(d)). Six hydrogen bonds and an electrostatic and hydrophobic interactions contributed to the binding energy. CYS54 interacted with genistein in atom O with a distance of 3.04 by a hydrogen bond. Asp27, Asp56, Glu53, and Tyr38 interacted with atom H of genistein with a hydrogen bond. Ile39 and Ser43 interacted with the aromatic ring of genistein by Pi-sigma and Pi-donor hydrogen bonds, respectively. At the same time, Glu57 was bound to a heterocyclic ring of genistein by Pi-anion.

**Figure 2:**
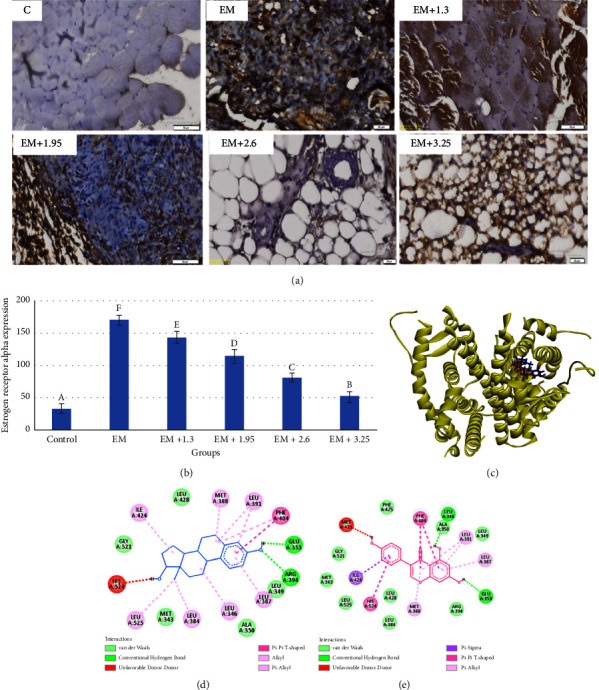
Estrogen receptor-*α* expression in various doses treatment of genistein in endometriosis mice peritoneal tissue. (a) Immunohistochemical profiles of genistein treatment in endometriosis mice model, (b) estrogen receptor-*α* expression levels, (c) the binding sites of estradiol and genistein in estrogen receptor-*α*, (d) 2D structure of estradiol-estrogen receptor-*α*, and (e) 2D view of genistein-estrogen receptor-*α* complex.

Endometriosis is a disease that depends on hormone homeostasis such as estrogen. Immunohistochemical profiles of ER-*α* are described in Figure 2(a). Genistein improved the ER-*α* profiles and expression levels. In endometriosis peritoneal tissue, ER-*α* was higher than that in normal mice. Genistein treatment with varied concentrations reduced the ER-*α* in mice peritoneal tissue. Genistein with a dose of 3.25 mg/day improved ER-*α* as low as that in control mice (Figure 2(b)). Molecular docking of both estradiol and genistein to estrogen receptor-*α* protein revealed that the binding sites of estradiol and genistein were in the same position (Figure 2(c)). The 2D views performed the same interaction type, including hydrogen bond, hydrophobic interaction, and van der Waals force. Estradiol as a control for ER-*α* generated lower binding energy than genistein (Table 2). Several active sites were identified on estradiol and genistein, including GLU353, PHE404, LEU346, MET388, LEU391, ILE424, and LEU387. A similar active site indicated that genistein blocked ER-*α* competitively and might substitute estradiol as a substrate.

## 4. Discussion

Endometriosis, a female reproductive disorder characterized by abnormal growth of the endometrium, is closely associated with elevated levels of estrogen and VEGF (Vascular endothelial growth factor) [2, 3]. The heightened estrogen levels contribute to processes such as angiogenesis, inflammation, and cellular movement and invasion [1, 5, 6]. The present study delves into the significant impact of genistein on reducing VEGF and estrogen receptor-*α* expression in the peritoneal tissue of endometriosis mice models. This aligns with previous research where compounds like resveratrol demonstrated preventive effects on endometriosis by inhibiting prostaglandin synthesis, thus promoting anti-inflammatory responses, acting as antioxidants, and inducing cell apoptosis [6].

In addition to genistein, other bioactive compounds have exhibited promising effects in preventing chronic endometriosis, contributing to a diverse landscape of potential therapeutic interventions. Notably, epigallocatechin gallate, a polyphenolic compound found in green tea, has demonstrated multifaceted mechanisms in hindering the progression of endometriosis. Studies have indicated its effectiveness through antiangiogenic, anti-inflammatory, and antioxidant properties, underscoring its potential as a versatile therapeutic agent [20, 21]. Furthermore, its interference with nicotine metabolism adds an additional layer of complexity to its impact on endometriosis, suggesting a comprehensive approach in addressing the diverse pathways implicated in the disorder.

Quercetin, another flavonoid with well-established antioxidant and anti-inflammatory properties, has emerged as a potential regulator of endometriosis invasion and proliferation. This compound has been reported to exert its effects by modulating the activity of cyclin D, a key regulator of the cell cycle. By influencing cyclin D, quercetin may play a crucial role in controlling the unregulated cell growth characteristic of endometriosis, presenting a targeted approach to mitigate the invasive nature of the condition [22]. In contrast, black rice anthocyanins, belonging to the flavonoid group, have been identified as inhibitors of apoptosis mechanisms associated with endometriosis. Specifically, their action involves the inhibition of caspase-3, a pivotal enzyme involved in the programmed cell death process. This unique property suggests that black rice anthocyanins may contribute to the maintenance of cell survival in the context of endometriosis, potentially influencing the longevity and persistence of endometrial cells outside the uterus.

This study emphasizes the significance of angiogenic and estrogen receptor-*α* (ER-*α*) pathways in endometriosis therapy. Elevated estradiol levels are known to activate ER-*α*, promoting proinflammatory and inflammatory cytokines. The in vivo study conducted in this research revealed that a daily dose of genistein at 3.25 mg significantly reduced ER-*α* expression. In silico docking studies further indicated that genistein competitively inhibited ER-*α*, akin to estrogen. The structural similarity between genistein and estradiol, both featuring two benzene rings forming the 3-phenylchromen-4-one structure, suggests a potential interference with the estradiol-ER-*α* interaction. However, it is noteworthy that the binding energy of estradiol and ER-*α* was lower than genistein, indicating a tighter interaction, although the inhibition mechanism may not be significantly affected by binding energy alone [22, 23]. Various factors, including the type of interactions, the number of hydrogen bonds, hydrophobic interactions, and van der Waals forces, contribute to the overall binding energy [18, 19, 24].

The inhibitory effects of genistein on VEGF, as evidenced in both in vivo and in silico studies, represent a significant stride in understanding its potential therapeutic role in managing endometriosis. Vascular endothelial growth factor (VEGF) is a key player in angiogenesis, a process intricately linked to the pathogenesis of endometriosis. Angiogenesis, the formation of new blood vessels, is a crucial aspect of endometrial implantation and the establishment of ectopic endometrial tissue, making it a prime target for therapeutic interventions.

Numerous studies have underscored the pivotal role of VEGF blockers in impeding angiogenesis and, consequently, mitigating the progression of endometriosis. This study aligns with existing research by showcasing that genistein joins the ranks of compounds capable of hindering VEGF expression, thereby potentially disrupting the angiogenic processes associated with endometriosis. The significance of this lies not only in understanding the specific actions of genistein but also in recognizing its place within a broader spectrum of potential therapeutic agents for endometriosis.

The arsenal of VEGF inhibitors reported to date encompasses a diverse array of compounds, each with its unique mechanisms and potential applications in preventing chronic endometriosis. These include soluble truncated VEGF receptor, iodamin, endostatin, anginex, sorafenib, statin, lipoxin, parecoxib, pycnogenol, melatonin, resveratrol, metformin, and colchicine [25, 26]. The sheer variety of these inhibitors underscores the intricate nature of the molecular pathways involved in endometriosis and the need for a multifaceted approach in developing therapeutic strategies.

This comprehensive exploration into the inhibitory effects of genistein on VEGF not only provides valuable insights into its potential therapeutic mechanisms but also highlights the need for continued research into innovative therapeutic strategies for endometriosis.

## 5. Conclusion

The study revealed that the administration of genistein at a dosage of 3.25 mg per day had a positive effect in ameliorating the expression of VEGF-A and ER-*α* in the peritoneal tissue of an endometriosis mice model. In silico analysis demonstrated the inhibitory mechanism of genistein at the active sites of VEGF-A. Furthermore, genistein acted as a competitive inhibitor for ER-*α*, competing with estradiol as the native ligand. These findings provide further insights into the potential of genistein as a therapeutic agent in managing endometriosis by indicating its impact on key factors involved in the pathogenesis of the disease. Additionally, the in silico results enhance our understanding of the interactions between genistein and target molecules, paving the way for further research regarding compound design and more effective treatment strategies.

## Figures and Tables

**Table 1:** Interaction of genistein with VEGF-A protein.

Complex interactions	Binding energy (kJ/mol)	Interaction	Distance (A)	Category	Type
Genistein-VEGF-A	−274.3	A:CYS54:N—:10:O4	3.04	Hydrogen bond	Conventional hydrogen bond
		:10:H8—D:ASP27:OD1	2.01	Hydrogen bond	Conventional hydrogen bond
		:10:H9—A:ASP56:O	2.14	Hydrogen bond	Conventional hydrogen bond
		:10:H10—D:GLU35:O	2.30	Hydrogen bond	Conventional hydrogen bond
		:10:H10—D:TYR38:O	1.94	Hydrogen bond	Conventional hydrogen bond
		A:GLU57:OE1—:10	4.27	Electrostatic	Pi-anion
		D:SER43:OG—:10	2.99	Hydrogen bond	Pi-donor hydrogen bond
		D:ILE39:CA—:10	3.91	Hydrophobic	Pi-sigma

**Table 2:** The interaction of estradiol and genistein with estrogen receptor-*α* protein.

Complex interactions	Binding energy (kJ/mol)	Interaction	Distance (A)	Category	Type
Estradiol-estrogen receptor-*α*	−262	A:ARG394:NH2—:10:O2	2.61	Hydrogen bond	Conventional hydrogen bond
		:10:H24—A:GLU353:OE1	1.63	Hydrogen bond	Conventional hydrogen bond
		A:PHE404—:10	4.93	Hydrophobic	Pi-Pi T-shaped
		A:LEU346—:10	4.81	Hydrophobic	Alkyl
		A:MET388—:10	4.95	Hydrophobic	Alkyl
		A:LEU391—:10	4.77	Hydrophobic	Alkyl
		A:ILE424—:10	4.96	Hydrophobic	Alkyl
		:10:C11—A:LEU384	4.94	Hydrophobic	Alkyl
		:10:C11—A:LEU525	3.96	Hydrophobic	Alkyl
		A:PHE404—:10	5.38	Hydrophobic	Pi-alkyl
		:10—A:LEU387	5.03	Hydrophobic	Pi-alkyl
		:10—A:LEU391	4.73	Hydrophobic	Pi-alkyl

Genistein-estrogen receptor-*α*	−250	:10:H8—A:LEU346:O	2.48	Hydrogen bond	Conventional hydrogen bond
		:10:H9—A:GLU353:OE1	2.02	Hydrogen bond	Conventional hydrogen bond
		A:ILE424:CG2—:10	3.83	Hydrophobic	Pi-sigma
		A:PHE404—:10	5.21	Hydrophobic	Pi-Pi T-shaped
		A:PHE404—:10	5.13	Hydrophobic	Pi-Pi T-shaped
		A:HIS524—:10	5.78	Hydrophobic	Pi-Pi T-shaped
		:10—A:LEU387	4.71	Hydrophobic	Pi-alkyl
		:10—A:MET388	5.23	Hydrophobic	Pi-alkyl
		:10—A:LEU391	4.58	Hydrophobic	Pi-alkyl
		:10—A:LEU391	5.05	Hydrophobic	Pi-alkyl
		A:MET421:N—:10:H10	2.12	Unfavorable	Unfavorable donor-donor

## Data Availability

The data used to support the findings of this study are available from the corresponding author upon request.
